# Myelin Oligodendrocyte Glycoprotein (MOG) Antibody Diseases in Children in Central South China: Clinical Features, Treatments, Influencing Factors, and Outcomes

**DOI:** 10.3389/fneur.2019.00868

**Published:** 2019-08-08

**Authors:** Leilei Mao, Lifen Yang, Miriam Kessi, Fang He, Ciliu Zhang, Liwen Wu, Fei Yin, Jing Peng

**Affiliations:** ^1^Department of Pediatrics, Xiangya Hospital, Central South University, Changsha, China; ^2^Hunan Intellectual and Developmental Disabilities Research Center, Changsha, China

**Keywords:** myelin oligodendrocyte glycoprotein (MOG), optic neuritis (ON), demyelinating diseases, disease-modifying drugs, rituximab

## Abstract

**Background and purpose:** The clinical and radiological features of myelin oligodendrocyte glycoprotein antibody (MOG-Ab) diseases vary among the patients and studies. In addition, the clinical significance of MOG-Ab for the diagnosis, treatment, and prognosis is not yet established. Therefore, we aimed to evaluate the clinical, radiological, treatments and outcome features of MOG-Ab diseases in Central Southern part of China.

**Methods:** A retrospective study of children with MOG-Ab disease was carried out from January 2015 to October 2018. Demographics, clinical features, treatments, and outcomes were reviewed. Some of the clinical information was compared including the annualized relapse rates (ARRs) before and after treatment with disease-modifying drugs (DMDs).

**Results:** Twenty-five patients with MOG-Ab disease were recruited. The onset age ranged from 3 to 12.4 years old. 13 were females and 12 were males. The median follow-up period was 15 months (range 7–63). Most of the cases that aged ≤9 years presented with fever (47.4%), encephalopathy (47.4%), and lesions on white matter and/or deep gray matter (52.6%). While most of those aged above 9 years presented with optic neuritis (ON) (66.7%), and lesions on spinal cord and/or optic nerve (50%). Until the last follow-up, 10 (40%) cases had multiphasic courses while 15 (60%) had a monophasic course, and the mean follow-up time was statistically significant (10.67 vs. 31 months, *p* = 0.0001). DMDs such as rituximab (RTX) or/and azathioprine (AZP) or mycophenolate mofetil (MMF) were used at least once in 56% of the cases. The ARR before and after treatment was 2.4 and 0 respectively (*p* < 0.05). The median Expanded Disability Status Scale scores of our study were 0 (range 0–2). 96% (24/25) of the cases had a full recovery.

**Conclusions:** MOG-Ab disease among Chinese children share the same clinical characteristics with Caucasians. However, the Chinese children seem to have a better prognosis than Caucasians. There is an age-dependent phenotypes, as brain involvement is more frequently seen in children younger or equal to 9 years while ON and neuromyelitis optica spectrum disorders are commonly seen in children older than 9 years. DMDs, such as AZA, MMF or RTX, can reduce the ARR.

## Introduction

Myelin oligodendrocyte glycoprotein (MOG) is a glycoprotein that is localized on the outer surface of the myelin sheath and oligodendrocytes (1). It has been used to induce autoimmune encephalomyelitis in an animal model of multiple sclerosis (MS) (2). The MOG antibody (MOG-Ab) has been identified in pediatric patients with acquired demyelinating syndrome, particularly the acute disseminated encephalomyelitis (ADEM) (3–5). In addition to ADEM, this antibody has also been found in patients with other inflammatory demyelinating diseases (IDDs) such as clinically isolated syndrome (CIS), neuromyelitis optica spectrum disorders (NMOSDs), recurrent bilateral optic neuritis (ON), transverse myelitis (TM), and MS (2, 6).

Patients with MOG-Ab-associated demyelination seem to have unique clinical and radiological features. Nevertheless, the clinical significance of MOG-Ab for the diagnosis, treatment and prognosis is not yet established. The radiological findings associated with these Abs vary among patients and studies. Since its discovery, many cases have been reported in many countries, including China. However, the existing studies from China have very small sample sizes.

This study aimed to evaluate the clinical, radiological, treatments, and outcome features of MOG-Ab diseases. To our knowledge, this study contains the largest sample size in China to date.

## Subjects and Methods

We retrospectively reviewed the clinical data including the symptoms, physical examination and ancillary examination findings [magnetic resonance imaging (MRI), electroencephalographs (EEG), and cerebrospinal fluid (CSF)], treatments used, and their outcomes. This study included children who were aged 14 years old or younger with a definite diagnosis of MOG-Ab-positive with IDDs at Xiangya Hospital, Central South University. The cohort comprised patients admitted at our center from January 2015 to October 2018. The written and informed consent were obtained from the patients' parents.

For the better assessment of the clinical course of this condition we defined an acute episode as a new neurological deficit lasting <24 h (7). A relapse referred to any new central nervous system (CNS) symptom/sign lasting >24 h in the absence of other causes that was supported clinically or radiologically (8). The criteria for IDDs, including ADEM, NMOSD, CIS, and MS, were in accordance with those of the International Pediatric MS Study Group (9). ADEM-ON was defined as at least one episode of ON after ADEM (10). Any immune-mediated CNS demyelinating disorder not falling into the aforementioned categories was classified as an uncategorized CNS demyelination. All the patients were followed up, and data were collected and analyzed. Patients with a follow-up duration of <5 months were excluded. The dosages and durations of medications used during the acute phase and disease-modifying drugs (DMDs) were reviewed. Complete recovery was defined as a disappearance of clinical symptoms and brain lesions. Almost complete recovery was defined as a disappearance of clinical symptoms with persistence of brain lesions. Partial recovery was defined as a persistence of both clinical symptoms and brain lesions. We compared the annualized relapse rates (ARRs) before and after treatment with DMDs in relapsing patients. The Expanded Disability Status Scale (EDSS) score (rates from 0 to 10) was used to estimate the disability outcome at the last follow-up.

Serum samples from all patients were tested for anti-MOG immunoglobulin G (IgG) and anti-AQP4 IgG by the Chinese branch of the Euroimmun Medical Diagnostic Laboratory using a fixed cell-based indirect immune-fluorescence test (IIFT) employing BIOCHIPs (EUROIMMUN AG, Luebeck, Germany). HEK293 cells transfected with full-length human MOG and AQP4 isoform M1 were used in the anti-MOG and anti-AQP4 IIFTs, respectively. Serum specimens were collected from these patients to test MOG-IgG during the acute attacks or while undertaking follow-up visits from January 2018.

The statistical analyses were performed using SPSS version 19.0 (SPSS Inc., Chicago, IL, USA). Continuous variables, such as the age and follow-up time, were analyzed with an independent *t*-test. Categorical variables were analyzed with the chi-square test or Fisher's exact test. *P* < 0.05 (two-sided) was considered significant.

## Results

A total number of 54 patients were diagnosed to have IDDs from January 2015 to October 2018 (NMOSD 25 patients; ADEM 17 patients; MS 3 patients; CIS 1 patient; ON or TM 5 patients; Others 3 patients). And the serum samples of all 54 patients were tested for MOG-Ab in which only 25 had positive results.

### The Clinical Characteristics

The present study comprised of 25 cases who had first demyelinating symptoms from the age of 3 to 12.4 years. 12 were males, and male to female ratio was 1:1.08. Table 1 summarizes the patients' clinical information. Nineteen cases were ≤ 9 years old at the onset of the disease (ranged 3–8.8 years with median of 5.75 years). Table 2 summarize the demographic and clinical characteristics of those groups.

**Table 1:** The demographic and clinical details.

**Case number**	**Sex**	**Onset age, year**	**Follow-up time, months**	**Re-lapse numbers**	**Onset symptoms**	**Clinical manifestation**	**Prodro-mal symp-toms**	**Lesion location in MRI at onset**	**Lesion location during the follow-up period**	**Immuno- suppressive treatment**	**EDSS at last follow-up**
1	F	4.8	24	7	Fever, Headache, Encephalopathy	1st episode of ADEM (acute encephalitis), 7 episodes of rON	None	Subcortical white matter, cervical spinal cord	Optic nerve, cervical spinal cord	mPSL, IVIG, RTX, AZP	0
2	M	10.9	13	0	Myelitis	LETM	URI 2 weeks before onset	Cervical spinal cord	Cervical spinal cord	mPSL, IVIG	0
3	F	3.25	12	0	Myelitis, encephalopathy, seizure	ADEM	None	Subcortical white matter, spinal cord	Subcortical white matter	mPSL, IVIG, RTX	0
4	F	5.8	15	0	Encephalopathy, hemiparesis, external ophthalmoplegia	ADEM	None	Subcortical white matter, cerebellum, cerebral peduncle, spinal cord	Subcortical white matter, cerebellum, cerebral peduncle	mPSL, IVIG	0
5	M	6.7	15	0	Hemiparesis, seizure, fever, myelitis, encephalopathy	ADEM	None	Corpus callosum, subcortical white matter	Corpus callosum, subcortical white matter	mPSL, IVIG	0
6	F	7.2	20	0	Seizure, speech delay, poor cognition	ADEM	None	Subcortical white matter, corpus callosum, thalamus, basal ganglia, and cerebellum	N/A	mPSL	0
7	M	11.5	16	0	BON	BON	URI 2 weeks before onset	subcortical white matter, thalamus	Subcortical white matter, thalamus	DEX, mPSL, IVIG	0
8	M	3	51	1	Headache, unsteadily gait	1st episode of headache and unsteady gait, 2nd episode of unsteady gait	None	Subcortical white matter, cerebellum	Subcortical white matter, basal ganglia, medulla, and cerebellum	mPSL, IVIG, mycophenolate mofetil	0
9	M	10.5	23	1	Myelitis	NMOSD	Mumps 20 days before onset	Subcortical white matter, spinal cord	Spinal cord	mPSL, IVIG, RTX	0
10	M	5.75	12	0	Seizure	CIS	None	Subcortical white matter, white matter adjacent to the posterior horn of lateral ventricle	None	mPSL, IVIG	0
11	F	5.2	13	0	Fever and encephalopathy	ADEM	None	Subcortical white matter, thalamus and basal ganglia, cerebellum, optic nerve, spinal cord	Subcortical white matter, thalamus and basal ganglia, cerebellum, optic nerve, spinal cord	IVIG, DEX, mPSL	0
12	M	6.4	14	0	Myelitis, headache, encephalopathy	ADEM	None	Subcortical white matter, basal ganglia, optic nerve, spinal cord	Subcortical white matter, basal ganglia, Optic nerve, spinal cord	DEX, IVIG, RTX	0
13	F	5.5	25	2	BON	NMOSD	None	Subcortical white matter, thalamus, brain stem, optic nerve	Subcortical white matter, thalamus, brain stem, optic nerve	mPSL, IVIG, RTX	0
14	F	4.5	63	2	Fever, headache	3 episodes of fever and headache	None	Subcortical white matter	Subcortical white matter	mPSL, IVIG, RTX, AZP	0
15	F	7.1	42	2	Encephalopathy	1st episode with ADEM, 2nd episode with UON (L), 3rd episode with headache and psychological and behavioral abnormalities	None	Subcortical white matter	Subcortical white matter, optic nerve	mPSL, IVIG, RTX, AZP	0
16	M	11.8	17	0	Headache, BON	NMOSD	None	Cervical spinal cord	None	mPSL	0
17	F	4.75	8	0	BON	BON	None	Thalamus	Thalamus, right optic nerve	mPSL, IVIG, RTX	0
18	F	8.75	9	0	Fever, Headache	1st episode with fever and headache	None	Subcortical white matter, thalamus	Subcortical white matter, thalamus, brain stem	mPSL, IVIG	0
19	M	8.8	44	3	BON	NMOSD	None	Subcortical white matter	Subcortical white matter	mPSL, IVIG, RTX	2
20	F	7	12	2	Unsteady gait, speech delay	1st episode with ADEM, 2nd episode with headache and myelitis	None	Subcortical white matter	Subcortical white matter, basal ganglia and corpus callosum	mPSL, IVIG, RTX	0
21	F	8.5	8	0	Encephalopathy seizures	ADEM	None	None	Subcortical white matter, and basal ganglia	mPSL, IVIG, RTX	0
22	M	5.4	7	0	Fever, headache	1st episode with fever and headache	None	Cortex, white matter adjacent to the posterior horn of the lateral ventricle	Cortex, white matter adjacent to the posterior horn of the lateral ventricle	mPSL, IVIG	0
23	M	11.6	12	4	UON (R)	5 episodes of rON	None	Optic nerve	Optic nerve	mPSL, AZP	0
24	F	5.75	36	1	Encephalopathy and seizures	MDEM	Fever 2 months prior onset	Subcortical white matter, hippocampi	Subcortical white matter, Hippocampi	mPSL, IVIG	0
25	M	12.4	12	0	Fever, encephalopathy and seizures	ADEM	None	Cortex	Cortex and thalamus	DEX, mPSL, IVIG, RTX	0

*M, male; F, female; AZP, azathioprine; ADEM, acute disseminated encephalomyelitis; BON, bilateral optic neuritis; CIS: clinically isolated syndrome; DEX, intravenous dexamethasone; EDSS, expanded disability scale score; IVIG: intravenous immunoglobulin; L, left; LETM, longitudinally extensive transverse myelitis; MOG-Ab, myelin oligodendrocyte glycoprotein antibody; MS, multiple sclerosis; mPSL, methylprednisolone pulse therapy; MDEM, multiple disseminated encephalomyelitis; MMF, mycophenolate mofetil; N/A, not applicable; NMOSD, neuromyelitis optica spectrum disorders; PSL, oral prednisone; PE, plasma exchange; R, right; rON, recurrent optic neuritis; RTX, rituximab; URI, upper respiratory infection; UON, unilateral optic neuritis*.

**Table 2:** Demographics, clinical characteristics and MRI findings.

**Item**	**All patients (%)**	**Age ≤9 (%)**	**Age >9 (%)**
Number	25	19	6
Female: male	13:12	13:6	0:6
Median age, range (years)	6.6, 3–12.4	5.75, 3–8.8	11.6, 10.6–12.4
**Onset symptoms**			
Fever	11 (44%)	9 (47.4%)	2 (33.3%)
Encephalopathy	9 (36%)	9 (47.4%)	0
ON	7 (28%)	3 (15.8%)	4 (66.7%)
Headache	7 (28%)	5 (26.3%)	2 (33.3%)
Seizures	6 (24%)	5 (26.3%)	1 (16.7%)
Myelitis	5 (20%)	3 (15.8%)	2 (33.3%)
Hemiparesis	2 (8%)	2 (10.5%)	0
Unsteady gait	2 (8%)	2 (10.5%)	0
**Disease spectrum**			
ADEM or MDEM or ADEM-ON	10 (40%)	9 (47.4%)	1 (16.7%)
NMOSD	6 (24%)	4 (21%)	2 (33.3%)
ON or rON or LETM	4 (16%)	1 (5.3%)	3 (50%)
MS or CIS or Others	5 (20%)	5 (26.3%)	0
Prodromal symptoms	4 (16%)	1 (5.3%)	3 (50%)
Initial lesions on MRI			
White matter and/or deep gray matter	10 (40%)	10 (52.6%)	0
White matter and spinal cord/optic nerve and/or others	8 (32%)	6 (31.6%)	2 (33.3%)
Spinal cord and/or optic nerve	3 (12%)	0	3 (50%)
Gray matter and/or white matter	2 (8%)	1 (5.3%)	1 (16.7%)
Cerebellum and/or others	2 (8%)	2 (10.5%)	0

*ADEM, acute disseminated encephalomyelitis; CIS, clinically isolated syndromes; LETM, longitudinally extensive transverse myelitis; MDEM, multiple disseminated encephalomyelitis; MS, multiple sclerosis; NMOSDs, neuromyelitis optica Spectrum disorders; ON, optic neuritis; rON, recurrent optic neuritis*.

The initial clinical presentations were as follows: 11 (44%) cases had fever, 9 (36%) had encephalopathy, 7 (28%) had ON (6 bilateral and 1 unilateral), and 7 (28%) had headache. The top four onset symptoms in the group of patients ≤ 9 years were fever (47.4%), encephalopathy (47.4%), headache (26.3%) and seizures (26.3%). The top four onset symptoms in the group of patients older than 9 years were ON (66.7%), fever (33.3%), headache (33.3%) and myelitis (33.3%). Disease onset was preceded by infection in 4 patients; upper respiratory tract infection (*n* = 3) and mumps (*n* = 1) which occurred 2 weeks to two months before the onset of the initial attack.

**Figure 1:**
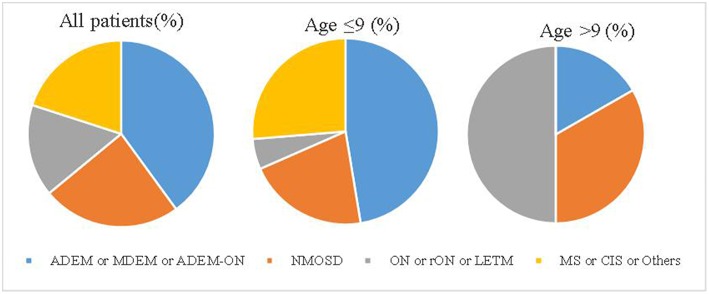
The disease spectrum of each group. ADEM, acute disseminated encephalomyelitis; CIS, clinically isolated syndrome; LETM, longitudinally extensive transverse myelitis; MS, multiple sclerosis; MDEM, multiple disseminated encephalomyelitis; NMOSDs, neuromyelitis optica spectrum disorders; ON, optic neuritis; rON, recurrent optic neuritis.

The median follow-up period was 15 months (range 7–63). Until the last follow-up, 10 (40%) patients had multiphasic courses. The median time between the first and second episodes was 10 months (range 3–32). The symptoms during the relapses were different from those of initial presentation. Regarding the initial phenotypes, 48% (12/25) of the cases presented with ADEM, 24% (6/25) with ON and 28% (7/25) with other CNS demyelination. Finally, according to the IDD criteria, 10 patients were diagnosed with ADEM (8 with ADEM, 1 with multiphasic disseminated encephalomyelitis (MDEM), and 1 with ADEM-ON), 6 (24%) with NMOSD (4 patients had a monophasic course while 2 had multiphasic courses), 1 with MS, 1 with CIS, and 7 with uncategorized CNS demyelination (4 patients with monophasic or recurrent ON or longitudinally extensive transverse myelitis (LETM). ADEM was diagnosed more in the group of patients ≤ 9 years old while those aged above 9 years presented more with monophasic or recurrent ON or LETM (Figure 1).

### The Ancillary Examination Results

Lumbar punctures were performed for 24 patients at first episode. Intracranial pressure was measured in only 20 cases as it was difficult for the other 4 cases. Five of those 20 cases had increased intracranial pressure (>200 mm H_2_O in patients number 1, 2, 14, 21, and 24). Elevated levels of CSF proteins and cell counts were found in 25% (6/24) of the cases. Two patients were found to have positive CSF oligoclonal bands (OCB) (Table 3).

**Table 3:** The laboratory findings at first episode.

**Case number**	**Sex**	**ICP, mmH2O**	**CSF protein, mg/L**	**CSF WBC, × 106/L**	**OCB**	**Serum MBP-Ab**	**Serum AQP4-Ab**	**CSF anti-NMDAR**	**ESR, mm/h**	**Other circulating antibodies**
1	F	230	140	180	–	–	–	–	23	A-TG/A-TPO+
2	M	205	330	20	–	–	–	–	44	A-TPO+
3	F	130	160	0	–	–	–	–	37	A-TPO+
4	F	150	330	50	–	–	–	–	91	A-TPO+
5	M	150	550	150	+	–	–	–	110	–
6	F	N/A	420	16	–	–	–	–	N/A	A-TG+
7	M	175	220	4	–	–	–	–	3	–
8	M	N/A	460	14	N/A	–	–	–	120	A-TG/A-TPO+
9	M	165	200	4	N/A	–	–	–	9	–
10	M	N/A	240	10	–	–	–	–	79	A-TPO+
11	F	82	540	0	–	–	–	–	67	A-TPO+
12	M	68	550	16	–	–	–	–	37	–
13	F	110	460	46	–	–	–	–	87	–
14	F	205	210	18	–	–	–	–	37	–
15	F	110	100	0	–	–	–	–	20	–
16	M	N/A	N/A	N/A	N/A	N/A	N/A	N/A	71	–
17	F	140	140	14	N/A	–	–	N/A	54	–
18	F	175	110	24	–	–	–	–	16	–
19	M	195	380	14	+	–	–	N/A	33	–
20	F	N/A	250	2	–	N/A	N/A	+/–	19	–
21	F	300	480	18	–	–	–	++	81	–
22	M	155	640	86	N/A	–	–	–	116	Anti-Ro-52+
23	M	190	140	0	–	–	–	–	11	–
24	F	280	410	0	–	–	–	–	14	–
25	M	150	300	50	–	–	–	+	84	A-TP0+

*M, male; F, female; AQP4-Ab, Aquaporin-4 antibody; anti-NMDAR, anti-N-methyl-D-aspartate receptor; A-TPO, thyroid peroxidase antibody; A-TG, thyroglobulin antibody; CSF, cerebral spinal fluid; ESR, erythrocyte sedimentation rate; ICP, intracranial pressure; MBP, myelin basic protein; N/A, not applicable; OCB, oligoclonal bands; WBC, white blood cell; “+”, positive; “–”, negative*.

Four patients (patient number 19, 20, 21, and 25) were found to have anti-N-methyl-D-aspartate receptor (anti-NMDAR) antibodies once. Patient number 19 was found to have anti-NMDAR antibodies on the second episode, of which manifested with headache and seizures. The other 3 patients were found to have antibodies on the first episodes. However, none of the four cases met the diagnostic criteria for anti-NMDAR encephalopathy. 66.7% (16/24) of the cases had an elevated erythrocyte sedimentation rate (ESR) (Table 3). Thirteen of the 17 patients whom underwent EEG examinations had abnormal findings. Twelve patients had diffuse background slowing (delta or theta) activity and/or generalized or predominantly frontotemporal slow wave activity, and one displayed both slow wave and epileptic activity. Six children received antiepileptic drugs; levetiracetam (*n* = 1) and oxcarbazepine (*n* = 5), and none of them had seizures during the follow-up period.

**Figure 2:**
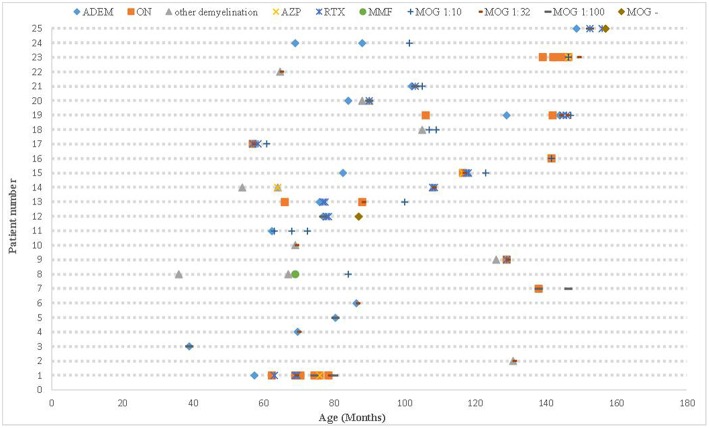
The clinical course, MOG-Ab titers, and DMDs of all patients. ADEM, acute disseminated encephalomyelitis; AZP, azathioprine; DMDs, disease-modifying drugs; MOG-Ab, myelin oligodendrocyte glycoprotein antibody; MMF, mycophenolate mofetil; ON, optic neuritis; RTX, rituximab.

All 25 cases had positive MOG antibodies in serum but only 6 out 23 cases whom underwent CSF screening had positive results. The relationship between the patients' phenotype and the MOG titer is shown in Figure 2. We traced the MOG-Ab titers of 12 cases (7 with a monophasic course and 5 with multiphasic courses). The MOG-Ab titers were low and unchanged during remission for 58.3 and 25% of the cases, respectively. Conversely, the titers were high during remission and relapse for one case each. Only 2 patients who had a monophasic course had a negative MOG-Ab titer at the last follow-up. The MOG-Ab titer was positive even 17 months after the disappearance of the symptoms.

All cases but number 21 had abnormal brain or spinal cord MRI at the onset. The abnormal findings were: sixteen cases had an increased signal on T2-weighted MRI or fluid-attenuated inversion recovery on the brain only (six had lesions on the white matter only, five on the white matter and deep gray matter and/or cerebellum/optic nerve, two on the gray matter and/or white matter, one on the thalamus only, one on the optic nerve only, and one on the subcortical white matter, thalamus, brain stem, and optic nerve), six on both brain and spinal cord (four on the subcortical white matter and spinal cord and two on the white matter, deep gray matter, optic nerve and spinal cord), and two on the cervical spinal cord only. Case number 21 had no lesion on the brain MRI during the first 10 days of the disease onset, but they were observed 20 days later on the subcortical white matter and basal ganglia. The abnormal signal locations on the MRI were split into five categories based on the symptoms (white matter and/or deep gray matter, white matter and spinal cord/optic nerve and/or others, spinal cord and/or optic nerve, gray matter and/or white matter, and cerebellum and/or others). All cases with an abnormal signal on the white matter and/or deep gray matter were ≤ 9 years, and all spinal cord and/or optic nerve abnormalities were found in patients older than 9 years (Table 2). When the MRIs from the follow-up period were compared with the initial ones, three children had an asymptomatic new abnormal signal on the brain, the lesions of three children (case number 10, 16, and 21) disappeared, and the lesions of 21 cases decreased regardless of whether relapse occurred.

### The Treatments and Outcomes

Acute attacks were treated with methylprednisolone pulse therapy (mPSL) (15–30 mg/kg per day for 5 days), followed by prednisone (1 mg/kg, tapered off within 1–2 months or more) at least once in 24 patients and with intravenous immunoglobulin (IVIG) (0.4 g/kg per day for 5 days) at least once in 22 patients. Other treatments included either methylprednisolone therapy (PSL) (3–5 mg/kg per day for 3–5 days) or intravenous dexamethasone (DEX) (20 mg/m^2^ per day for 5 days) followed by prednisone in one case each as well as acyclovir and/or antibiotics (*n* = 24) as empirical treatment for the suspected CNS infection.

The information regarding the treatment outcomes was available for 49 episodes. 14 documented attacks were treated only with mPSL, 26 with both mPSL and IVIG, 1 with both PSL and IVIG, and 1 with both DEX and IVIG. Conversely, 3 attacks were treated only with PSL, and 4 were not treated at all. Complete or almost complete recovery from acute attacks was noted after 43 episodes (27 were treated with both steroids and IVIG, 10 with mPSL only, 3 with PSL only and 3 were not treated at all) and partial recovery was noted after 3 attacks (2 were treated with mPSL only while 1 with both mPSL and IVIG). Notably, symptoms flared up after withdrawal or tapering of steroids at least once in 4/10 patients.

DMDs were prescribed once in 14 (56%) cases. These included RTX (2 × 750 mg/m^2^, days 1 and 15) in 10 cases, azathioprine (AZP) in 1 case (1 mg/kg), both AZP and RTX in 2 cases (case number 1 and 14) and mycophenolate mofetil (MMF) in 1 case (case number 8). Table 4 shows the efficacy of different DMDs in cases with a multiphasic course. The time from initial attack to administration of DMDs was 11 (3–54) months. The ARRs before and after treatment were 2.4 (0.67–8.22) and 0 (0–3.43), respectively (*P* < 0.05). 7/9 cases did not relapse after DMDs treatment. For those relapsed, one patient used AZP initially (ARR was 2.4 before the treatment and 0.24 after the treatment) and then changed to RTX (ARR became 0 after the treatment). The other patient used RTX and then changed to AZP because of an elevated ARR (ARR 4.8 before the treatment and 5.33 after the treatment).

**Table 4:** Efficacy of different disease-modifying drugs in patients with multiphasic course.

**Disease-modifying drug**	***N***	**Disease duration before treatment (m)**	**Duration of therapy (m)**	**ARR before treatment**	**ARR during treatment**
Rituximab	5	3	18	8	0
		11	12	2.4	1.09
		54	7	0.67	0
		39	4	1.23	0
		6	4	6	0
Azathioprine	1	7.3	2.7	8.22	0
Mycophenolate mofetil	1	33	16	0.73	0
Total, median(range)	7	11 (3–54)	7.5 (2.7–18)	2.4 (0.67–8.22)	0 (0–1.09)^*^

ARRs, annualized relapse rates;

* *p-value obtained by independent t-test; m, months*.

At the last follow-up, all patients were alive, and the final EDSS scores were low (ranging 0–2). The visual acuity (VA) of the 9 patients with a history of ON recovered (VA>1.0) except for the case number 19 who had several black spots in the field of vision (EDSS range 2). The median observation time was 21 months (range 6–42). 96% (24/25) of the cases recovered completely.

### Comparison Between Cases With Monophasic and Multiphasic Courses

The mean follow-up time of the patients with multiphasic course was 34.1 months (range 12–63) vs. 13.3 months (range 7–20) for the patients with a monophasic course (*P* = 0.0001). The median time between the first and second episodes was 10 months (range 3–32). The median time between the second and third episodes was 1.5 months (range 1–44). The sex, age at onset, clinical manifestations, ancillary examinations, locations of lesions on MRI and response to treatment did not differ between the two groups. We also compared variables between the cases with NMOSD (*n* = 4) and those with other relapsing IDDs (*n* = 6). And we found that sex, age at onset, phenotypes, seizures, onset of lesion on MRI, ARR and EDSS during follow-up did not differ (Table 5).

**Table 5:** Comparison between patients with NMOSD and those with other relapsing IDDs.

	**NMOSD**	**Other relapsing IDDs**	**All relapsed patients**
Number	4	6	10
Female: male	2:2	4:2	6:4
Age at onset, y, median (range)	8.0 (5.5–10.5)	6.1 (3–11.5)	6.85 (3–11.5)
Relapse episodes, median (range)	3 (2–4)	3 (2–7)	3 (2–7)
**Phenotype at initial attack**, ***n*** **(%)**			
ADEM	1 (25%)	3 (50%)	4 (40%)
ON	3 (75%)	1 (16.7%)	4 (40%)
Other demyelinating diseases	0	2 (33.4%)	2 (20%)
**Phenotypes in all attacks**, ***n*** **(%)**			
ADEM	4/12 (33.3%)	4/22 (18.2%)	8/34 (23.5%)
ON	6/12 (50%)	11/22 (50%)	17/34 (50%)
Other demyelinating diseases	2/12 (16.7%)	7/22 (31.8%)	9/34 (26.5%)
**Initial lesions on MRI**, ***n*** **(%)**			
White matter and/or deep gray matter	2 (50%)	3/6 (50%)	5 (50%)
White matter and spinal cord/optic nerve and/or others	2 (50%)	1 (16.7%)	3 (30%)
Spinal cord and/or optic nerve	0	1 (16.7%)	1 (10%)
Gray matter and/or white matter	0	0	0
Cerebellum and/or others	0	1 (16.7%)	1 (10%)
ARR during follow-up, median (range)	0 (0–1.09)	0 (0–3.43)	0 (0–3.43)
EDSS at last follow-up, median (range)	0 (0–2)	0	0 (0–2)
Seizures, n (%)	0	1 (16.7)	1 (10%)

*ARRs, annualized relapse rates; ADEM, acute disseminated encephalomyelitis; EDSS; Expanded Disability Status Scale; IDDs, inflammatory demyelinating diseases NMOSDs, neuromyelitis optica spectrum disorders; ON, optic neuritis*.

## Discussion

The first evidence for the role of MOG-Ab IgG as a biological marker in children was identified by O'Connor et al. (11) with MOG-Ab-associated demyelination in a subgroup of ADEM patients. In subsequent studies, MOG-Ab IgG was detected in a subset of ADEM, NMOSD, monophasic and recurrent ON and TM patients as well as in those with demyelinating syndromes overlapping with anti-NMDA receptor encephalitis. The clinical features were different between adults and children, and Hacohen and Brenda Banwell proposed that children with MOG-Ab were frequently Caucasian (12). A recent study from Australia showed the clinical courses, treatments and outcomes of 59 cases (33 pediatric patients) with relapsing MOG-Ab-associated demyelination, of whom 73% were Caucasian (13). This study summarizes the clinical features of 25 MOG-Ab-positive Chinese children aged below 14 years.

We found no gender difference between affected males and females (male: female = 1:1.08), of which is similar to previous report (12). Fever and encephalopathy were the most frequent onset symptoms in the group of children ≤ 9 years old, whereas ON was the most frequent onset symptom in the group of children older than 9 years. Consistent with our results, other studies demonstrated the same age-dependent phenotypes, with brain involvement more frequently seen in younger children while ON and NMOSD are common in older children (>9 years) (14) and adults (15). We further found that ADEM was the most frequent initial clinical presentation followed by ON. Peschl et al. performed a literature review and compared all studies that analyzed the presence of MOG-Ab in IDDs in which they found that ADEM was the most frequent initial clinical presentation associated with MOG-Ab, followed by ON (16). Four cases in our study (case number 19, 20, 21, and 25) had concomitant anti-NMDAR antibodies in at least one attack. When we compared our 4 current cases with those in our initial study of anti-NMDAR encephalitis (17), we found a reduced mean time to full recovery (1 vs. 4 months) and higher relapse rate (50 vs. 2%). Fan et al. (18) found that the symptoms of MOG-Ab diseases coexisting with anti-NMDAR encephalitis were frequently milder than those of typical anti-NMDAR encephalitis cases, which is in agreement with the findings of this study. Therefore, patients with anti-NMDAR encephalitis should also be tested for MOG-Ab. Our study showed that 6 cases had seizures and 13 cases had abnormal EEGs of which is similar to the recent study by Hennes et al. which showed five out of 34 cases had seizures (19).

Ten cases had multiphasic course up to the last follow-up, and the mean age of these children was 6.45 years. Similar to previous studies, 33.8–54% of children experience clinical relapses (20, 21). However, Hamid et al. (22) reported that the relapse frequency of MOG-Ab-positive cases was 93% with a long observation period (≥8 years) in adults. Compared with that of the children who had monophasic courses, the mean follow-up time was significantly different in the children who had multiphasic courses (31 vs. 10.67 months, *p* = 0.0001). Therefore, the recurrence rate might increase with the extended follow-up time.

The relationship between the MOG-Ab titers and clinical disease activity remains an area of active investigation, with a recent report suggesting that a high MOG-Ab titer (≥1:1280) predicted a recurrent non-MS course with a sensitivity of 46% and specificity of 86% (20). A large nationwide French study of 197 adults with MOG-Ab (15) observed that the titers were higher at relapse than in remission, but only two patients became seronegative. Similar to these studies, the MOG-Ab titer changed to a higher mean in a relapsing patient and to a lower or constant mean in patients in remission (10/11, 90.9%), and only 2 patients became seronegative. Few patients had increased ICP, CSF protein levels, WBC counts and ESRs, implying the presence of intense intrathecal inflammation during the active phase. According to these findings, MOG-Ab disease in its acute stage should be cautiously differentiated from CNS infection and vasculitis (23). A positive CSF OCB is not common in the patients with MOG-Ab as only 10% of the cases had positive results. However, 36% of the cases had concomitant circulating autoantibodies or autoimmune diseases of which is peculiar from the findings of patients with MS.

Hacohen et al. (12) proposed that children with MOG-Ab might present with four MRI patterns: (1) multifocal hazy/poorly marginated lesions involving both the gray and white matter and typically involving the middle cerebellar peduncles; (2) spinal cord and/or optic nerve involvement with a normal intracranial appearance or non-specific white matter lesions; (3) extensive and periventricular white matter lesions resembling a “leukodystrophy-like” pattern; and (4) cortical encephalitis with leptomeningeal enhancement. According to this proposal and the symptoms of the children in our study, the abnormal signal locations upon MRI were split into the following five categories: white matter and/or deep gray matter, white matter and spinal cord/optic nerve and/or others, spinal cord and/or optic nerve, gray matter and/or white matter, and cerebellum and/or others. The results showed an abnormal signal in the white matter and/or deep gray matter in all patients ≤ 9 years old and abnormal spinal cord and/or optic nerve signals in all patients older than 9 years (Table 2). However, these categories did not predict recurrence, and all lesions decreased or disappeared by the last follow-up.

Our study provides a detailed retrospective analysis of the effects of various DMDs in our patients. Similar to previous studies (24–26), treatment of acute attacks with mPSL and IVIG was effective in most patients. However, 4 out of 10 cases had symptoms that flared up after withdrawal or tapering of prednisone. IVIG was not used alone in our patients. Previous studies showed that breakthrough attacks under AZP were linked to the drug-specific latency period and lack of treatment with oral steroids (24). Treatment with RTX resulted to disease stabilization in some reported cases but was followed by early relapses in several cases; end-of dose relapses occurred 9–12 months after the first infusion (24). The recent multicenter European study (23) showed that AZP, MMF, and RTX were all effective at reducing relapses, although the median EDSS score remained unchanged. In our study, RTX, AZP, and MMF usage for those with multiphasic courses were also effective at reducing relapses (ARR from 2.4 to 0). Seven out of nine cases who used DMDs in addition had no more relapses (median follow-up 6.6 months).Of importance, we found that RTX was effective at reducing the MOG-Ab titer. Consequently, RTX seems to be effective at reducing not only the ARR but also the MOG-Ab titer in a short follow-up time.

In previous studies, severe visual impairment or functional blindness was present at the last follow-up in 36% of all cases, and impaired ambulation due to paresis or ataxia was found in 25% of adult patients (27). However, in our study, the VA of the seven cases out of eight with a history of ON was almost recovered (VA>1.0) at the last follow-up. 10 cases with multiphasic course in our study had the median EDSS score of 0 (follow-up time was 1–5.25 years). A total of 102 children with relapsing MOG-Ab disease who were all Caucasians had a median EDSS score of 1 or 2.5 in different subgroups (follow-up time was 3–9.1 years) (23). Additionally, 23 Chinese cases with relapsing MOG-Ab disease had a median EDSS score of 1 at the last follow-up (1–8.92 years) (28). The median EDSS score of 13 children from the Partners Pediatric MS Center at Massachusetts General Hospital (29) was 2 (mean follow-up time was 4.88 years), and the score for 9 children from Japan (30) was 0 (4 children had multiphasic course, follow-up time was 8–21 years). Altogether, these findings indicate different prognosis between races and suggest that pediatric Xanthoderms with MOG-Ab disease may have better outcomes than Caucasians.

Our study was limited by a small sample size and retrograde nature of making diagnosis of MOG-positive diseases since all MOG-Ab assay were performed from January 2018.

## Conclusions

Despite the limitations, this *post-hoc* evaluation and analysis of previously collected and published data allowed us to make important observations about MOG-Ab disease in Chinese children who share the same clinical characteristics as Caucasians but have a better prognosis. We have identified age-dependent phenotypes; encephalopathy and lesions on the white matter and/or deep gray matter are more prominent in younger children while lesions on the spinal cord and/or optic nerve are more common in older children. Most of the patients presented with ADEM followed by ON. 40% of the cases had relapses. Treatment of acute attacks with mPSL and IVIG were effective in most of the patients. DMDs, such as AZA, MMF, or RTX can reduce the ARR. Despite of multiple relapses, all patients except one had full recovery.

## Data Availability

The datasets generated for this study are available on request to the corresponding author.

## Ethics Statement

This study was carried out in accordance with the recommendations of the research ethics committee of Xiangya Hospital with written informed consent from all subjects. All subjects gave written informed consent in accordance with the Declaration of Helsinki. The protocol was approved by the research ethics committee of Xiangya Hospital.

## Author Contributions

LM and JP conceived the study. LY, CZ, and FH provided the clinical information and LM checked. LY, LW, and JP analyzed the data. LM drafted the initial manuscript which was edited by FY, MK, and JP.

### Conflict of Interest Statement

The authors declare that the research was conducted in the absence of any commercial or financial relationships that could be construed as a potential conflict of interest.
